# Comparative Study of Two Box H/ACA Ribonucleoprotein Pseudouridine-Synthases: Relation between Conformational Dynamics of the Guide RNA, Enzyme Assembly and Activity

**DOI:** 10.1371/journal.pone.0070313

**Published:** 2013-07-29

**Authors:** Jean-Baptiste Fourmann, Anne-Sophie Tillault, Magali Blaud, Fabrice Leclerc, Christiane Branlant, Bruno Charpentier

**Affiliations:** Laboratoire Ingénierie Moléculaire et Physiopathologie Articulaire (IMoPA), Unité Mixte de Recherche Centre National de la Recherche Scientifique - Université de Lorraine, Biopôle de l’Université de Lorraine, Vandœuvre-lès-Nancy, France; Univ. of Edinburgh, United Kingdom

## Abstract

Multiple RNA-guided pseudouridine synthases, H/ACA ribonucleoprotein particles (RNPs) which contain a guide RNA and four proteins, catalyze site-specific post-transcriptional isomerization of uridines into pseudouridines in substrate RNAs. In archaeal particles, the guide small RNA (sRNA) is anchored by the pseudouridine synthase aCBF5 and the ribosomal protein L7Ae. Protein aNOP10 interacts with both aCBF5 and L7Ae. The fourth protein, aGAR1, interacts with aCBF5 and enhances catalytic efficiency. Here, we compared the features of two H/ACA sRNAs, Pab21 and Pab91, from *Pyrococcus abyssi*. We found that aCBF5 binds much more weakly to Pab91 than to Pab21. Surprisingly, the Pab91 sRNP exhibits a higher catalytic efficiency than the Pab21 sRNP. We thus investigated the molecular basis of the differential efficiencies observed for the assembly and catalytic activity of the two enzymes. For this, we compared profiles of the extent of lead-induced cleavages in these sRNAs during a stepwise reconstitution of the sRNPs, and analyzed the impact of the absence of the aNOP10–L7Ae interaction. Such probing experiments indicated that the sRNAs undergo a series of conformational changes upon RNP assembly. These changes were also evaluated directly by circular dichroism (CD) spectroscopy, a tool highly adapted to analyzing RNA conformational dynamics. In addition, our results reveal that the conformation of helix P1 formed at the base of the H/ACA sRNAs is optimized in Pab21 for efficient aCBF5 binding and RNP assembly. Moreover, P1 swapping improved the assembly of the Pab91 sRNP. Nonetheless, efficient aCBF5 binding probably also relies on the pseudouridylation pocket which is not optimized for high activity in the case of Pab21.

## Introduction

Pseudouridine (Ψ) is one of the most common modified ribonucleotide found in functional regions of non-coding RNAs [1]–[8]. Site-specific post-transcriptional isomerization of uridine (U) residues into Ψ residues is catalyzed by a variety of specific RNA:Ψ-synthases [9]–[12]. In bacteria, tRNAs and rRNAs are exclusively modified by mono-polypeptide enzymes, which both recognize and modify the RNA [13]. As an alternative to protein stand-alone enzymes, numerous catalysts achieve U to Ψ conversions in eukaryal rRNAs and UsnRNAs, and in archaeal rRNAs. They consist in multiple distinct H/ACA ribonucleoprotein particles (RNPs). In eukaryotes, these are referred to as H/ACA small nucleolar (sno)RNPs, while in archaea, the corresponding designation is H/ACA sRNPs [14]. Each RNP comprises a unique non-coding RNA (snoRNA in eukaryotes or sRNA in archaea) and a common pseudouridine synthase (NAP57 or Dyskerin in vertebrates, Cbf5 in yeast, and aCBF5 in archaea). H/ACA RNPs also contain three other essential and evolutionary conserved core proteins, denoted Nop10, Gar1, and Nhp2 in H/ACA snoRNPs, and aNOP10, aGAR1, and L7Ae, respectively in H/ACA sRNPs [14]–[16]. In this RNP-based modification system, the RNA component functions as a guide, forming complementary base-pair interactions with a target RNA sequence in order to define the residue to be modified by the RNA:Ψ-synthase.

**Figure 1:**
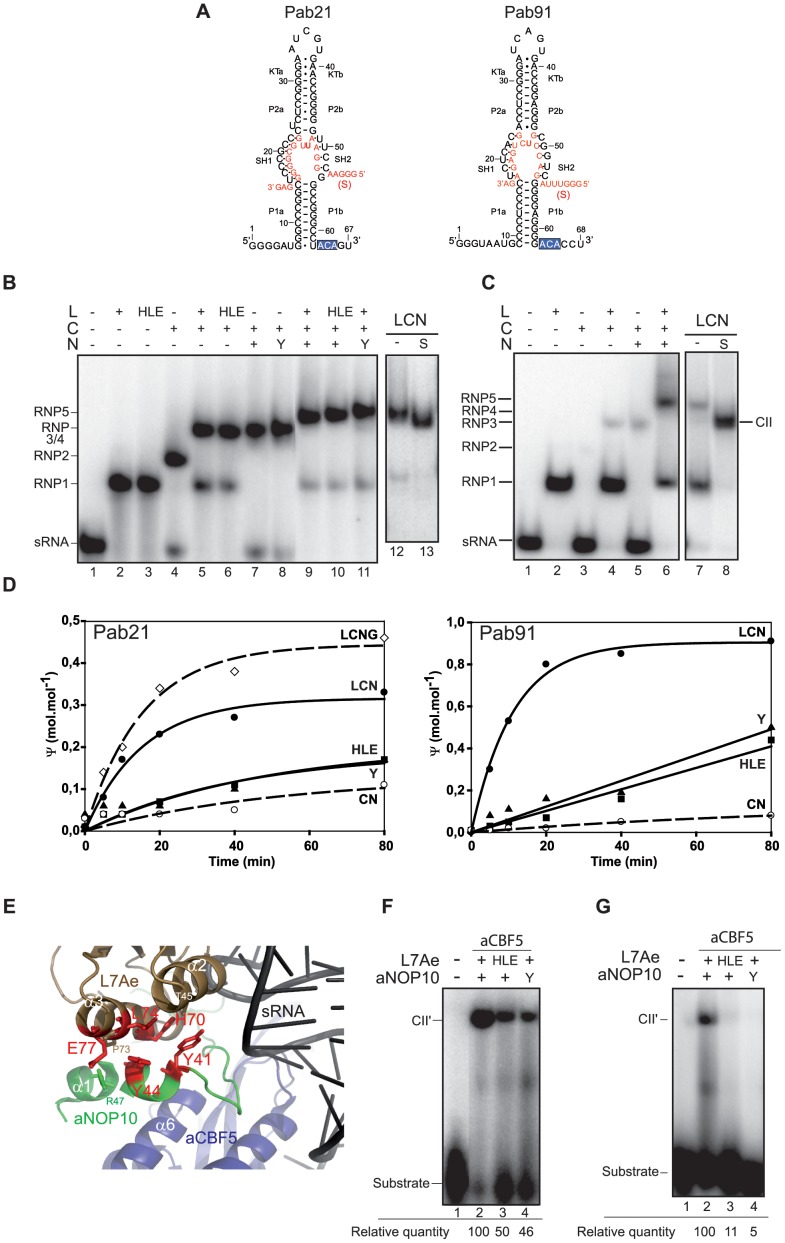
Effect of mutations of residues lying at the aNOP10–L7Ae interface on H/ACA sRNP assembly and activity. (**A**) Secondary structure model of *P. abyssi* Pab21 and Pab91 sRNAs and of the interaction between the sRNAs and their respective substrate RNA (S). The ACA sequence at the 3′ end is boxed. The strands of the various motifs are indicated: KTa and KTb of the apical K-loop motif, P2a and P2b of helix P2, and P1a and P1b of helix P1. Helices SH1 and SH2 are formed upon interaction of the substrate RNA with the two strands s1 and s2 of the pseudouridylation pocket. (**B** and **C**) Electrophoretic mobility shift assay (EMSA) of the various sub-complexes formed upon incubation of the radiolabeled sRNAs (50 fmol) Pab21 (**B**) or Pab91 (**C**) with various combinations of the wild-type and mutant proteins (200 nM each). (**D**) Time course analyses of the RNA-guided Ψ formation in the substrate RNAs, which were radiolabeled during *in vitro* transcription by incorporation of [α-^32^P]UTP for Pab21 substrate and of [α-^32^P]CTP for Pab91 substrate. Each substrate RNA was incubated at 65°C with the unlabeled guide sRNA, and the protein sets aCBF5–aNOP10 (CN), L7Ae–aCBF5–aNOP10 (LCN), or L7Ae–aCBF5–aNOP10–aGAR1 (LCNG). A mutant of L7Ae (triple mutant H70A/L74A/E77A, designated HLE), or a mutant of aNOP10 Y41A/Y44A (designated Y) were used for the reaction. After T2 RNase digestion, the amount of Ψ formation was estimated by 1D-TLC analysis. (**E**) Close-up view of the contact region between L7Ae (displayed in gold) and aNOP10 (in green). Residues conserved between *P. abyssi* and *P. furiosus* are indicated on the *P. furiosus* sRNP structure (PDB 2HVY). Residues in red were substituted by alanine. (**F** and **G**) Analysis by EMSA of the complexes formed between the wild-type and mutant Pab21 sRNP (**F**) and Pab91 sRNP (**G**) and their respective radiolabeled substrate RNA. The yield of complex CII’ is indicated below each lane; the percentage of sRNA present in each CII′ complex was estimated by radioactivity measurement. The yields are expressed relative to that obtained with wild type sRNP (set to 100).

The majority of information on the structure and function of H/ACA RNPs derives from experiments performed on archaeal particles. The H/ACA sRNAs identified in archaeal genomes share common features with eukaryotic snoRNAs, but differ in their heterogeneous architectures [17]–[21]. Archaeal sRNAs fold into structures comprising one, two, or as many as three units linked together by single-stranded ANA sequences (ACA being the most frequently observed), and incorporate a single-stranded ACA sequence at their 3′ ends (the ACA box). Each basic unit consists of an irregular hairpin which includes a lower helix P1, a large internal loop called a pseudouridylation pocket, an upper helix P2, and an apical loop. An apical K-turn or K-loop motif (KT) is present on top of helix P2 in all the archaeal basic units (as shown in Figure 1). Two sequences (s1 and s2) on both sides of the internal loop establish two distinct base-pair interactions (SH1 and SH2) with sequences flanking the target U in the substrate RNA. In such complexes, the U to be modified and its neighboring 3′ residue are unpaired, resulting in a three-helix junction around the exposed 5′-UN-3′ di-nucleotide [22]–[24].

Information on the role of the core proteins and the conserved RNA elements for sRNP assembly and activity has been obtained by studies performed with reconstituted particles [17], [18], [25], [26]. These experiments demonstrated that box H/ACA sRNPs can be assembled *in vitro* using recombinant proteins purified from *E. coli* and *in vitro*-transcribed guide sRNAs, and reconstituted sRNPs pseudouridylate substrate RNAs consisting of simple target oligoribonucleotides [17], [18], [27]. Only aCBF5 and L7Ae were found to interact directly and stably with the sRNA [17], [18]. L7Ae binds the apical KT, while aCBF5 requires the ACA tri-nucleotide for association with the sRNA. In the absence of sRNA, aCBF5 forms a heterotrimer via interaction with aNOP10 and aGAR1 [17], [18], [28]. Hence, binding of aCBF5 to the guide sRNA allows the recruitment of aNOP10 and aGAR1 to the particles. Although the RNA-guided activity of aCBF5 relies on its interaction with aNOP10, sub-sRNPs formed with the pair of proteins aCBF5–aNOP10 display low activity [18], [25], [29]. A fully-active sRNPs is only obtained upon addition of L7Ae [17], [18]. The fourth protein aGAR1 is not required for total modification of substrate RNA, but its presence enhances the rate of Ψ formation [18], by facilitating catalytic turnover [30], [31].

Crystal structures have been obtained for i) the RNA-free and -bound forms of L7Ae [32]–[34]; ii) aNOP10 [35]; iii) isolated heterodimeric and heterotrimeric complexes formed between the remaining core proteins, i.e. aNOP10–aCBF5 and aNOP10–aCBF5–aGAR1 [25], [28], [36]; and iv) a complete H/ACA sRNP including the four proteins and a single-hairpin H/ACA sRNA [37]. In the latter complex, L7Ae, aNOP10, and aCBF5 are stacked against each other, with aNOP10 sandwiched between the other proteins, and the three proteins together bind one side of the H/ACA sRNA. In accordance with biochemical data, i) aCBF5 is anchored to the ACA sequence by its PseudoUridine synthase and Archaeosine transglycosylase (PUA) domain, whereas its catalytic domain contacts the pseudouridylation pocket; ii) aNOP10 is bound to the catalytic domain of aCBF5; and iii) L7Ae is tightly associated with the apical KT. The structure also revealed two features not previously deduced from the biochemical studies: L7Ae contacts aNOP10, and several residues of aNOP10 contact the upper helix P2 of the sRNA. In the RNP, the overall sRNA helical axis is bent slightly, so that its concave side is presented to the proteins. This bent conformation is also evident in the recently solved 3D structure of the sRNP associated with the substrate RNA [31], [38], [39]. X-ray crystallography on aCBF5–aNOP10 and aCBF5–aNOP10–aGAR1 complexes suggested that aNOP10 could organize the catalytic center of aCBF5 [28], [36], and specific residues in aNOP10 were indeed found to influence aCBF5 activity [29]. The RNA binding activity of L7Ae was suggested to remodel the sRNA pseudouridylation pocket [26], and L7Ae was also proposed to play an essential role in determining the proper placement of the substrate RNA in the catalytic center [40], [41].

Here, we investigated the respective conformations in various sub-RNPs or in RNPs lacking the L7AeaNOP10 interaction of two sRNAs exhibiting different efficiencies in guiding pseudouridylation. The analyses were performed in solution by chemical probing and circular dichroism (CD) spectroscopy. Our data demonstrate that the dynamic RNA conformation within RNPs can be minutely explored by CD. Moreover, our results clearly show that the H/ACA sRNA conformation depends on helix P1, which represents an important determinant for the efficiency of H/ACA RNP assembly.

## Materials and Methods

### H/ACA sRNP Reconstitution, EMSA and *in vitro* pseudouridylation Assay

As previously described [27], sRNPs were reconstituted by incubation of *in vitro* transcribed ^32^P 5′ end-labeled sRNA (∼50 fmol) with purified recombinant proteins (200 nM each) in buffer D [150 mM KCl; 1.5 mM MgCl_2_, 0.2 mM EDTA and 20 mM HEPES (pH 7.9)] at 65°C for 10 min. Association of the sRNA present in the sRNP with its substrate RNA was analyzed by two approaches [18], [27]. In the first, complex CII was formed by adding the unlabeled oligoribonucleotide (2.5 pmol) representing the substrate RNA (S) to the sRNP assembling mix described above, in the presence of proteins aCBF5, aNOP10 and L7Ae (200 nM each). In the second, sRNPs were assembled under the same conditions, but by incubating unlabeled *in vitro* transcribed sRNA (2 pmol) with radiolabeled oligoribonucleotide S (150 fmol). Complexes were visualized by autoradiography following fractionation by non-denaturing polyacrylamide gel electrophoresis.

Conditions for time course analysis of the RNA:Ψ-synthase activities of the reconstituted sRNPs were as detailed previously [27]. Briefly, 4 pmol of unlabeled sRNA and 150 fmol of [α-^32^P]CTP or [α-^32^P]UTP labeled substrate RNA were mixed at 65°C with the various sets of protein. Aliquots were collected at several time points and the reaction was stopped by phenol-chloroform extraction followed by ethanol precipitation. The recovered substrate RNAs were digested with RNase T2. The resulting 3′-mononucleotides were fractionated by monodimensional thin layer chromatography, and the radioactivity in the resulting bands was quantified with a phosphorimager using ImageQuant software. The quantities of Ψ residues formed were determined by taking into account the total number of U residues in the sRNA’s substrate RNA.

### Chemical Probing

sRNPs were assembled on ^32^P-end labeled sRNA using the same conditions as described in the preceding paragraph. sRNAs were labeled at their 5′ ends using T4 polynucleotide kinase and [γ-^32^P]ATP. For lead (II) footprinting, the reactions were initiated by addition of 40 mM Pb(II) acetate (Merck) freshly prepared in sterile water and incubated for 2 min at room temperature. Reactions were stopped by addition of 0.1 M EDTA and precipitated with 1 ml of ethanol at –80°C. Next, a phenol-chloroform extraction was performed and the treated RNA samples were ethanol precipitated in the presence of 0.3 M sodium acetate and 2 μg total yeast tRNAs. After centrifugation, the pellet was washed with 80% ethanol, and the dried RNA pellets were dissolved in RNA loading dye [95% (v/v) formamide, 20 mM EDTA, 0.05% (w/v) each bromophenol blue and xylene cyanol]. RNA alkaline hydrolysis ladders (cleavage after each nucleotide) were generated by incubating the labeled RNA in 1 M sodium carbonate at pH 9 for 3 min at 96°C. In order to generate RNase T1 ladders (cleavage after each guanosine), the labeled RNA was incubated in 1 M sodium hydroxide citrate in the presence of 2 µg tRNAs for 5 min at 65°C, and then treated with 1 U of RNase T1 for 10 min at 65°C. The cleavage products were separated on 10% polyacrylamide (acrylamide:bis ratio 38∶1) gels containing 8 M urea, and visualized by phosphorimaging.

### Circular Dichroism Spectroscopy Analysis

The CD spectra were recorded using a Jobin-Yvon Mark VI circular dichrograph at a scan speed of 0.2 nm/s. Quartz spare split-compartment cuvettes with 0.435 cm path length per compartment were used and spectra were scanned in the range of 200–320 nm. The final protein and/or RNA concentration was 2 µM, and the assays were carried out at 25°C in 10 mM Tris-HCl (pH 7.5) containing 150 mM NaCl. The protein and RNA spectra alone were recorded by placing the relevant solution in one compartment of the cuvette and buffer solution in the other. Studies of the protein-RNA interaction and sub-sRNPs formation were achieved using a sequential analysis. First, the protein solution (aCBF5) was placed in one compartment and the sRNA in the second one, and CD spectra were recorded before and after mixing the cuvette contents. Proteins aNOP10 and L7Ae were then added sequentially, and after five minutes of mixing, the spectrum of each sub-sRNP was measured. Blanks were obtained for each spectrum or sequential interaction, and the values subtracted from the raw data. In each case, two spectra were averaged to increase the signal-to-noise ratio. The results are presented as normalized Δε values on the basis of the mean residue mass of 330 Da. Taking into account a sensitivity of δ(ΔA) = 10^−6^ for the apparatus, the nucleotide concentration and the optical path-length of the cuvette, measurements were obtained at a precision of δ (Δε) = +/−0.02 M^−1^.cm^−1^ per nt.

### Molecular Dynamics (MD) Simulations on Pab21 and Pab91 RNA Hairpin Models

The Pab21 and Pab91 RNA hairpin models, Pab21P_1_ and Pab91P_1_ respectively, were built by assembling a canonical A-form RNA helix corresponding to the lower stem (P1) with a terminal loop mimicking the internal loop of the H/ACA sRNA, and the ACA box. The terminal loop was extracted from the 23S rRNA structure of *Deinococcus radiodurans* (PDB ID: 1J5A): it corresponds to the dodecaloop found at positions 2187 to 2198 (5′-AAAAAUCACUUU-3′) which best fits the size (11-mer or 12-mer) and sequence (proportion of purines and pyrimidines) of the peudouridylation pockets from Pab21 and Pab91. The RNA models were assembled using Mc-Sym [42]. They were then prepared for molecular dynamics simulations using the CHARMMing web interface [43], including the following steps: energy minimization, solvation within a cubic box with explicit solvent (TIP3P water model) and neutralization of the RNA net charge by the addition of sodium ions (34 Na^+^). The full system of nearly 20,000 atoms was equilibrated for 0.25 ns before a production run of 10 ns. The simulations were performed in the NVT ensemble at 298 K, using the CHARMM27 force field [44] and particle-mesh Ewald (PME) for the treatment of the electrostatic interactions (for more details on the algorithms and tools used in CHARMM, see [45]).

The MD trajectories were analyzed using: (1) the 3DNA software [46] to calculate the base-pair and base-pair step parameters, and (2) the Curves+ software [47] to calculate the base-pair helical parameters and bending of the helical axis (from the prior 3DNA analysis). All the simulations were performed using cloud computing from the Stratuslab services [48].

### Fluorescence Studies

The fluorescence studies were carried out by adapting the assay developed by Li and colleagues [41]. 2-AP and 5-FU labeled substrate RNA were purchased from Dharmacon (Thermo Fisher Scientific). All fluorescence experiments were recorded on an flx spectrofluorometer (SAFAS), at 55°C and each titration was measured in triplicate using different batches of guide RNA transcripts. The RNA–protein subcomplexes were preassembled in a specific binding buffer consisting of 300 mM NaCl, 25 mM MgCl_2_ and 180 mM Na citrate. The substrate RNA was added at a concentration of 1 µM and the molar ratio in reconstituted RNP subcomplexes was 1∶1∶5 for substrate RNA:guide RNA:proteins. The fluorescence intensity was recorded in kinetic mode until a plateau was reached, using an excitation wavelength of 325 nm and an emission wavelength of 366 nm. The samples were incubated for 10 minutes before each measurement.

## Results

### Two H/ACA sRNAs Which Exhibit Different Affinities for aCBF5 that do not Reflect their Different Efficiencies in Guiding Pseudouridylation

The guide sRNAs Pab21 and Pab91 from *Pyrococcus abyssi* (Figure 1A) share a single-hairpin conformation with an apical K-loop motif which is efficiently recognized by L7Ae, as shown by EMSA experiments (RNP1, lanes 2 in Figures 1B and 1C). Pab91 had been used in our first *in vitro* reconstitution assays of H/ACA sRNPs ([18]), and incubation of this sRNA with the three core proteins L7Ae, aCBF5 and aNOP10 (LCN), and the radiolabeled RNA substrate, lead to modification of ∼80% of the RNA substrate after 40 min of incubation (Fig. 1D). This robust activity is not correlated with the binding efficiency of the aCBF5 enzyme. Indeed, whereas Pab91 sRNA was completely shifted in presence of 200 nM L7Ae in a unique complex RNP1, only small amounts of RNA–protein complexes were formed upon incubation with 200 nM aCBF5 (RNP2, lane 3 in Figure 1C) alone or in stoichiometric combination with L7Ae (RNP3, lane 4 in Figure 1C) or with aNOP10 (RNP4, lane 5 in Figure 1C). In the presence of the three proteins, a complex of lower mobility RNP5 was observed, but only a fraction of the sRNA is shifted in this complex as it co-exists with complex RNP1 (lanes 6 and 7 in Figure 1C). The Pab91 sRNA was totally displaced in a unique RNP, only in the presence of an excess of RNA substrate (complex CII, lane 8 in Figure 1C).

In contrast to Pab91, Pab21 formed large amounts of RNA–protein complexes (RNP2 lane 4, RNP3 lane 5, RNP4 lane 7, and RNP5 lane 9 in Figure 1B). Nevertheless, the fully assembled Pab21 RNPs LCN and LCNG showed low activity, with less than 50% of the RNA substrates modified in an 80 min single-turnover reaction (Figure 1D).

### Substitution of Amino Acids Present in the aNOP10L7Ae Interface Impairs Activity of the H/ACA sRNPs

Amino acids in aNOP10 and L7Ae that lie at the interface between the two proteins were substituted by alanines in order to disrupt the interaction between the two proteins. In the sRNP crystal structure [37], aNOP10 helix α1 contacts L7Ae helix α3 (Figure 1E). In this interaction, aNOP10 residues Y41 and Y44 stack against a hydrophobic surface formed by L7Ae residues T45, A69, H70, P73, and L74. In addition, L7Ae residue E77 is proposed to form an ionic interaction with aNOP10 residue R47. Pab21 and Pab91 sRNPs were reconstituted by incubation of wild type proteins aCBF5, aNOP10, and L7Ae with the *in vitro* transcribed *P. abyssi* sRNAs Pab21 or Pab91. The Ψ-synthase activities of these wild type sRNPs were compared with those of sRNPs carrying mutations in either L7Ae or aNOP10 (Figure 1D). Substitution in aNOP10 of the two tyrosines Y41 and Y44 (variant Y41A/Y44A), or substitution in L7Ae of the three residues H70, L74, and E77 (triple variant H70A/L74A/E77A) reduced the rate of substrate modification by the two sRNAs. As the CD spectra of the wild type and the alanine-substituted proteins were identical (data not shown), the observed effect of the mutations on sRNP activity was not due to perturbations in the folding of the mutant proteins. Also, as shown by EMSA experiments, the low activity is not associated with a lower amount of RNA–protein complexes (Figure 1B), as similar amounts of RNP1, RNP3, RNP4 and RNP5 complexes were obtained with the wild type and the variant proteins.

Previous work *in vitro* has shown that aCBF5 is able to modify position U55 in tRNAs in the absence of guide sRNA. This position is modified in *E. coli* tRNAs by TruB [12], [29]. Importantly, the presence of aNOP10 becomes crucial for Ψ55 formation in a tRNA lacking the CCA 3′ extension [29]. In the present study, we observed that the aNOP10 mutant Y41A/Y44A was able to stimulate the non-RNA-guided activity of aCBF5 to the same extent as the wild type aNOP10 (Figure S1). As L7Ae does not influence such non-RNA-guided activity [29], data obtained with the aNOP10 mutant show that the aNOP10–L7Ae interface is not involved in the non-RNA-guided activity. Hence, the important role played by aNOP10 residues Y41 and Y44 was only observed in the context of H/ACA sRNPs containing L7Ae.

### Interactions between aNOP10 and L7Ae Influence Substrate RNA Binding with the sRNP

As aNOP10 was crucial for the binding of the substrate RNA to aCBF5–aNOP10 sRNPs [18], we investigated the role played by residues at the aNOP10–L7Ae interface in substrate RNA binding to sRNPs assembled with proteins L7Ae, aCBF5, and aNOP10. EMSA was used to visualize complexes resulting from the interaction between sRNPs and an oligoribonucleotide representing their substrate RNA. This analysis showed that a retarded complex, designated complex CII′ [18], was formed upon incubation of the substrate RNA with a large excess of the unlabeled Pab21 sRNP (Figure 1F, lane 2) and Pab91 sRNP (Figure 1G, lane 2). Mutations in residues lying at the aNOP10–L7Ae interface lead to lower amounts (∼2-fold decrease) of CII′ for sRNP Pab21 and to barely detectable CII′ for sRNP Pab91 (Figure 1F and 1G, lanes 3–4). These data support the relative effects of the variant proteins on sRNPs activities (Figure 1D).

### Interactions between aNOP10 and L7Ae Influence Substrate RNA Conformation within the Particle

We adapted the procedure developed by Li and co-workers [41] to analyze the conformation of the substrate RNA within the sRNP. This approach was only applied to the wild-type and the variant Pab21 RNPs for which the CII′ complex was detectable. The assay is based on the use of a substrate RNA, in which the nucleotide immediately 3′ to the target U is substituted by 2-aminopurine (2-AP). In case of the Pab21 substrate RNA, the corresponding residue is a U at position 11 (Fig. 1A). The intensity of the fluorescence emitted by this residue is correlated to its stacking state in the RNA structure; specifically, the intensity is highest when 2-AP is not stacked. Hence, the fluorescence intensity represents an ideal probe for analyzing conformational changes occurring in the substrate RNA following its binding to the sRNP. As controls, we verified that substitution of residue U11 by all of the other nucleotides had no significant effect on the efficiency of pseudouridylation, and that replacing it with 2-AP had no effect on the amount of CII′ complex formed (data not shown). In order to prevent pseudouridylation, the target U was substituted with 5-fluorouridine (5-FU).

**Figure 2:**
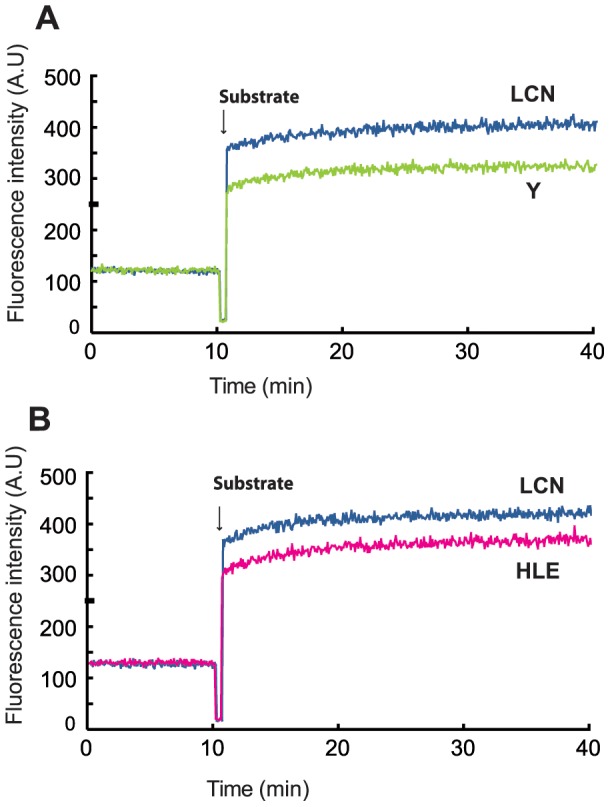
Effect of mutations in proteins L7Ae and aNOP10 on substrate RNA positioning within the sRNP. Fluorescence intensity was monitored while Pab21 guide RNA bound to aCBF5, aNOP10 and L7Ae was titrated with 2AP-labeled substrate RNA. Comparison of the fluorescence intensity profiles of titration by the wild type proteins (dark blue trace) to the profiles recorded with mutant proteins aNOP10 Y41A/Y44A (green trace) (**A**), and with L7Ae H70A/L74A/E77A (pink trace) (**B**). All proteins were present at 5× molar excess relative to the RNA to ensure full binding.

Addition of the substrate RNA to the preassembled wild type and mutant aCBF5–aNOP10–L7Ae–Pab21 complexes lead to a marked increase in the fluorescence intensity (Figure 2). Mutations introduced into the aNOP10–L7Ae interface conducted to a lower level of fluorescence intensity compared to the signal recorded with the wild-type RNP suggesting a distinct conformation of the substrate RNA. The potential role of the aNOP10–L7Ae interface in substrate docking was also probed by monitoring 2-AP fluorescence intensity, by studying the effect of sequential addition of the proteins on a preassembled substrate RNA–Pab21 complex (Figure S2). We concluded from these fluorescence data that the interaction between aNOP10 and L7Ae influences the conformation of the substrate RNA within box H/ACA RNPs.

### Chemical Probing of Sub-sRNPs

**Figure 3:**
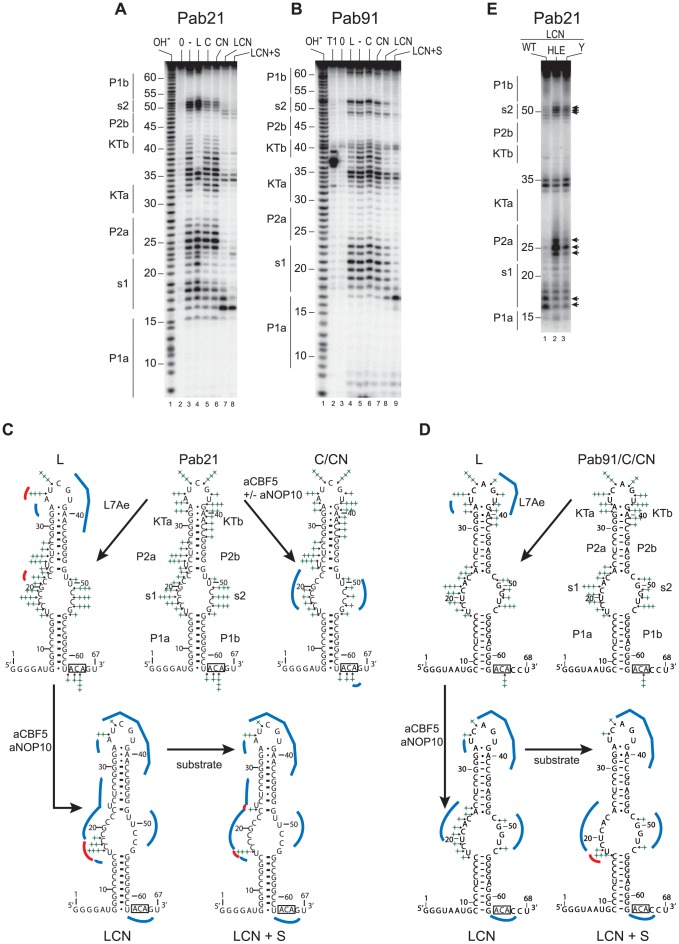
Chemical probing of sub-complexes of the Pab21 sRNP. (**A** and **B**) Footprinting of proteins L7Ae, aCBF5 and aNOP10 on sRNAs Pab21 and Pab91. Reactions with lead were carried out on 5′ ^32^P end-labeled Pab21 (panel **A**) or Pab91 (panel **B**). Samples were fractionated on 10% polyacrylamide denaturing gels containing 8 M urea. The protein set used for assembly of the probed protein–RNA complexes is indicated on top of each lane: L7Ae (L), aCBF5 (C), aCBF5–aNOP10 (CN), and L7Ae–aCBF5–aNOP10 (LCN). Probing was also performed on the complex obtained in presence of L7Ae–aCBF5–aNOP10 and an excess of the substrate RNA (LCN+S). Lanes OH^–^ and T1 correspond, respectively, to an alkaline hydrolysis ladder, and RNase T1 digestion ladder obtained under denaturing conditions. (**C** and **D**) Summary of probing data. Positions of the phosphodiester bonds cleaved upon incubation with lead of the naked sRNA and of the various sub-complexes L, C, CN, LCN, and LCN +S are indicated by stars on the secondary structure model of sRNA Pab21 (panel **C**) and Pab91 (panel **D**). The number of stars is proportional to the efficiency of the cleavages. Regions that were protected from cleavage are highlighted by blue lines and regions that displayed an increase in lead-induced digestion are highlighted by red lines. (**E**) Probing of the LCN complexes assembled with the mutant proteins of the aNOP10–L7Ae interaction, indicated with the same code as in Figure 1. The differences in cleavage products obtained with the wild-type and mutant LCN sub-complexes are indicated with arrows along the autoradiogram.

In order to analyze the individual contributions of the core proteins L7Ae, aCBF5, and aNOP10, as well as that of the aNOP10–L7Ae interaction to the sRNP conformation in solution, we used Pb^2+^ which typically induces cleavages in the RNA phosphate backbone of single-stranded regions, allowing the detection at high sensitivity of conformational changes in RNA molecules [49]. We used sRNAs Pab21 and Pab91 radiolabeled at their 5′-end for the probing experiments (Figures 3A and 3B). Following formation of the various sub-complexes of the sRNPs in the conditions used for EMSA (Figures 1B and 1C), we analyzed the accessibility of the sRNA to hydrolysis induced by Pb (II) acetate. By comparison with the cleavage profiles obtained with the sRNAs in the absence of proteins (Pab21 lane 3 in Figure 3A and scheme in Figure 3C; Pab91 lane 5 in Figure 3B and scheme in Figure 3D), the presence of L7Ae (lanes 4 in Figure 3A and Figure 3B, schemes L in Figures 3C and 3D) lead to strong protection against cleavages occurring in the apical K-loop motif (KTa and KTb sequences). These data are in accord with the binding of L7Ae to the K-loop motif. In addition to this protective effect, L7Ae RNA binding also resulted in an enhancement in the efficiency of lead cleavage in the apical loop between residues A34 and U35 in Pab21.

In the aCBF5–Pab21 complex (lane 5 in Figure 3A, scheme C in Figure 3C), the ACA box was found to be less accessible to lead-induced cleavage. This result was expected, as the PUA domain of aCBF5 binds the ACA motif and the lower stem P1 [25], [31], [37], [38], [40], [50]. We also observed a protection against cleavages between residues C17, C18, C19, G20, C21, C22 and C23 of strand s1, and residues U50, C51, C52, and C53 of strand s2. These protections may correspond to a footprint of the catalytic domain of aCBF5, which is known to sit against the pseudouridylation pocket [31], [38], [50].

In the presence of the aCBF5–aNOP10 heterodimer (lane 6 in Figure 3A, scheme CN in Figure 3C), the cleavage pattern was the same as that observed in the presence of aCBF5 alone.

The pattern of cleavages observed for the naked Pab91 was unchanged in the presence of aCBF5 or aCBF5–aNOP10 (compare lane 5 with lanes 6 and 7 in Figure 3B). This observation was not surprising, as the very low amounts of complexes observed by EMSA in Figure 1E would not allow detection of the footprint of aCBF5 on this sRNA.

Analysis of the complexes formed in the presence of L7Ae, aCBF5, and aNOP10 (lane 7 in Figure 3A and lane 8 in Figure 3B, scheme LCN in Figures 3C and 3D), revealed only a limited number of cleavages for the two sRNAs, which occurred in the apical loop and in strand s1. For the LCN Pab21 RNP, a strong cleavage occurred at the junction of the pseudouridylation pocket with helix P1. Hence, the presence of aNOP10 together with the two RNA binding proteins aCBF5 and L7Ae, lead to significant changes in the accessibility of the probe to the sRNA. These changes in probe accessibility are likely related to conformational changes occurring upon formation of contacts between aNOP10 and L7Ae, as were observed within the crystal structure of the fully assembled sRNP [31], [37], [38]. Remarkably, the probing signature characteristic of the L7Ae–aCBF5–aNOP10–Pab21 complex was not observed in the presence of mutants of either L7Ae or aNOP10 (lanes 2 and 3, respectively in Figure 3E). Indeed, lead still induced cleavages of the s2 and P2a strands of the mutant sRNPs, while the enhanced cleavages at the helix P1a–s1 junction were not observed.

Finally, we probed the Pab21 and Pab91 sRNAs within the CII complexes obtained in the presence of an excess of substrate RNA (Figures 1B lane 13 and 1C lane 8). For the two sRNAs, we observed a faster electrophoretic mobility for these complexes as compared to the RNP5 complexes, suggesting an additional conformation change occurring upon substrate RNA binding. For Pab21, the strong reactivity at the helix P1a–s1 junction was preserved. However, the significant cleavage between residues C17 and C18 disappeared, whereas the linkage between residues C23 and U24 at the basis of helix P2 was slightly more exposed to the probe (Figure 3A, lane 8). Interestingly, the strong reactivity at the helix P1a–s1 junction was also observed in the CII complex formed with Pab91 (Figure 3B, lane 9).

### Conformational Change of the sRNA in Various Sub-sRNPs

**Figure 4:**
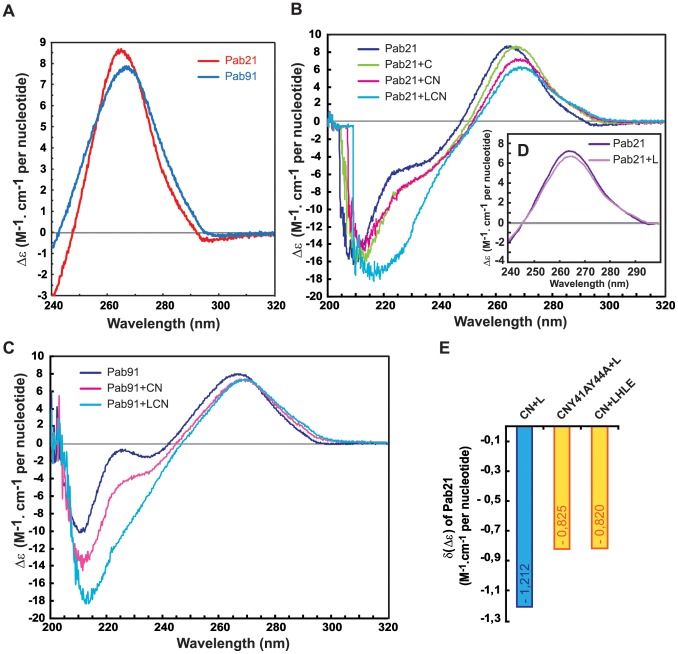
Different conformational changes of sRNA Pab21 and Pab91 are associated with the sequential binding of the sRNP proteins. (**A**) CD spectra of sRNAs Pab21 (red) and Pab91 (blue) scanned in the range 240 to 320 nm. (**B**) CD spectra of sRNA Pab21 obtained when protein aCBF5 and the sRNA are present in two separate cell compartments (dark blue) or after mixing the two compartments (green). Spectra obtained following sequential addition of proteins to the aCBF5–Pab21 complex: aNOP10 (magenta) added first and then L7Ae (light blue). (**C**) Same experiment as in panel B but with Pab91. (**D**) CD spectra of sRNA Pab21 obtained with the sRNA and L7Ae in separate compartments (dark purple) and after mixing (purple). (**E**) Quantification of the decrease in the peak amplitude of the specific RNA positive signal at the λ_max_ wavelength, generated exclusively by addition of L7Ae to different aCBF5–aNOP10–sRNA complexes. The resulting δ(Δε) values are displayed as a histogram for each complex. sRNP assembly was achieved by incubation of aCBF5–Pab21 with the mutant of L7Ae (H70A/L74A/E77A designated HLE) or the mutant of aNOP10 (Y41A/Y44A designated Y).

The chemical probing results suggested that the guide sRNA undergoes change in conformation during the sequential reconstitution of sub-sRNPs. We used far UV circular dichroism (CD) spectroscopy to analyze in more detail such conformational changes (Figure 4). This choice was motivated by the fact that CD spectroscopy is an established technique for studying RNA folding transitions in solution [51], and has been extensively used to probe protein-nucleic acid interactions, as two distinct wavelength windows provide information about the conformation of each of the partners in nucleoprotein complexes (for a review of this subject, see [52]). In the 200–240 nm range, CD spectra are dominated by signals from the amides of the protein backbone, although nucleic acids also make a significant contribution. In contrast, in the spectral region above 240 nm, nucleic acids give rise to a strong CD band compared with the relatively weak signal returned by protein aromatic side chains. This specific CD signal is characteristic of the level of nucleotide stacking (Δε value) and backbone conformation (λ_max_ of the band).

The CD spectra of free Pab21 and Pab91 displayed a positive signal in the 250–300 nm range with a maximal Δε value (Δε_ max_) at a wavelength referred as the λ_max_ wavelength (Figure 4A). These features indicated that a significant proportion of the sRNAs fold into an A-form helix. The λ_max_ of the signal for Pab21 (red line) and Pab91 (blue line) are respectively 263.8 nm and 265.6 nm. The lower λ_max_ measured for Pab21 suggests a more marked curvature in the global structure of this sRNA as compared to Pab91. Moreover, we measured a higher Δε_max_ for Pab21 compared to Pab91 (respective values of 8.54 and 7.87 M^−1^.cm^−1^ per nucleotide). This difference indicated the presence of a higher number of unstacked nucleotides in the Pab91 RNA helices compared to those of Pab21.

Addition of the proteins in a 1 to 1 stoichiometry with the RNA modified the CD spectra (Figure 4B and 4C), indicative of changes in the tertiary structure of the sRNA in the various sub-RNPs, but with preservation of the helical elements. Whereas the λ_max_ of the signal was unchanged upon L7Ae binding to Pab21, the amplitude of the peak was reduced (δ(Δε) = –0.78±0.02 M^−1^.cm^−1^ per nt, Figure 4D, purple line). The opposite occurred upon aCBF5 binding to Pab21, as the λ_max_ was displaced from 263.8 to 265.6 nm (±0.1 nm) although the amplitude of the peak was unchanged as compared to the spectrum obtained with the naked sRNA (Figure 4B, green line). These data indicate that binding of aCBF5 to Pab21 directly influenced the RNA bending compared to the binding of L7Ae, which induced a modification in the RNA local stacking. We did not observe modification in the CD spectrum of Pab91 upon aCBF5 binding (data not shown).

Binding of aNOP10 to the aCBF5–sRNA complex lead to both a displacement of the λ_max_ from 265.6 to 266.8 nm (±0.2 nm) for Pab21, and 265.6 to 267.6 nm (±0.2 nm) for Pab91, and a reduction in the Δε value (δ(Δε) = –1.28±0.02 M^−1^.cm^−1^ per nt for Pab21, and –0.57±0.02 M^−1^.cm^−1^ per nt for Pab91). Interestingly, Pab21 and Pab91 have comparable Δε value (7.2 M^−1^.cm^−1^ per nt) in the aNOP10-aCBF5-sRNA complex (Figure 4B and 4C, magenta lines). Hence, the presence of aNOP10 probably induced additional modifications in the conformation of the two sRNAs.

Addition of L7Ae to the aCBF5–aNOP10–Pab21 or –Pab91 complex had different outcomes. For Pab91, both the λ_max_ and the Δε values were kept unchanged whereas for Pab21, an additional increase in λ_max_ from 266.8 to 267.6 nm (±0.01 nm) was measured, but more remarkably, this effect was coupled with a further decrease in the RNA signal (δ(Δε) = –1.11±0.02 M^−1^.cm^−1^ per nt), compared to the spectrum of the aNOP10–aCBF5–sRNA complex (Figure 4B, light blue line)). The effect of L7Ae on the sRNA conformation is linked to the presence of aNOP10, as we observed that binding of L7Ae to the K-loop motif induced a smaller decrease in the RNA signal (δ(Δε) = –0.78±0.02 M^−1^.cm^−1^ per nt). In addition, CD spectra obtained after addition of the wild-type L7Ae to the aCBF5–aNOP10(Y41A/Y44A)–Pab21 complex and upon addition of the L7Ae mutant (H70A/L74A/E77A) to the wt aCBF5–aNOP10–Pab21 complex showed that the mutant sRNPs displayed lower δ(Δε) values than the wild type sRNP (–0.82 and –0.825 vs. –1.21±0.02 M^−1^.cm^−1^ per nt) (Figure 4E). In view of these data, we concluded that, as suggested by the lead cleavage experiments, substitutions which compromised the aNOP10–L7Ae interaction affected the conformation of the sRNA within the L7Ae–aCBF5–aNOP10 sRNP.

### Helix P1 is an Important Determinant for sRNA Conformation and RNP Assembly

**Figure 5:**
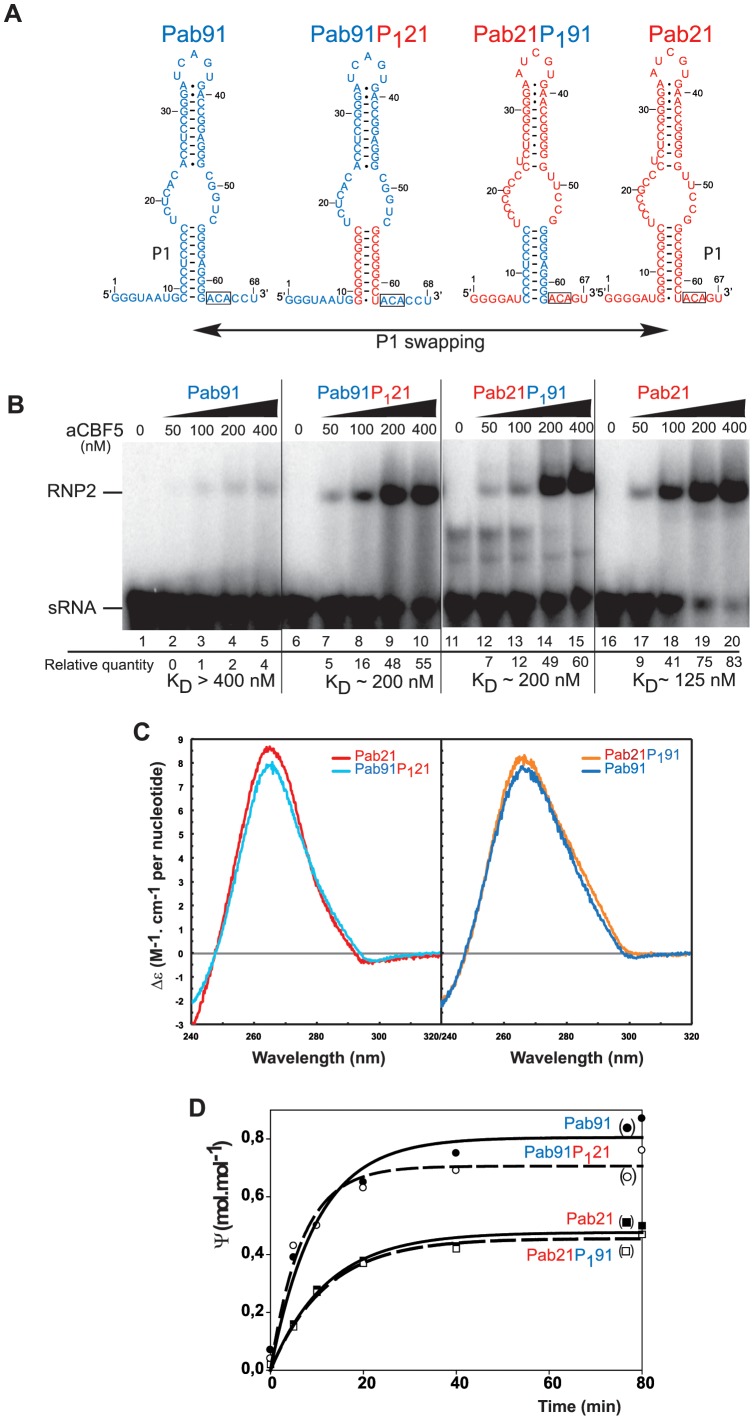
Swapping of helix P1 between sRNAs Pab21 and Pab91. (A) Scheme of the chimera obtained upon swapping the P1 basal helices. To obtain Pab91P_1_21, sequences spanning from positions 9 to 17 and sequences from positions 54 to 62 in Pab91 were respectively substituted by sequences of Pab21 spanning from positions 7 to 15 and from positions 54 to 62. To obtain Pab21P_1_91, sequences spanning from positions 7 to 15 and sequences from positions 54 to 62 in Pab21 were respectively substituted by sequences of Pab91 spanning from positions 9 to 17 and from positions 54 to 62. (**B**) EMSA of the sub-complex RNP2 formed upon incubation of the radiolabeled sRNAs (50 fmol) with various concentrations of the protein aCBF5 (50 to 400 nM) indicated on top of each lane. The amounts of radioactivity in the bands were estimated with the ImageQuant software. The percentage of RNA in each RNP was calculated from the radioactivity in each band relative to the total radioactivity in the lane. The estimated value of the apparent dissociation constant (K_D_) is indicated for each aCBF5-sRNA complex. (**C**) CD spectra of the parental Pab21 and Pab91 and the chimeric sRNAs. Experiments were performed as in Figure 4A. (**D**) Time course analyses of the Ψ formation by the LCN RNPs in the substrate RNAs of Pab21 (Pab21 and Pab21P_1_91) and of Pab91 (Pab91 and Pab91P_1_21). Experimental conditions were the same as in figure 1C. After T2 RNase digestion, the amount of Ψ formation was estimated by 1D-TLC analysis. The wild type sRNAs Pab91 and Pab21 are represented by solid lines and full symbols, while the chimeric sRNAs Pab91P_1_21 and Pab21P_1_91 are represented by dashed lines and empty symbols.

As shown by our comparative study on aCBF5 binding to Pab91 and Pab21 (Figure 1B & 1C), discrete elements present in the Pab21 sequence promote the formation of high levels of RNP2 complex, and the CD analyses indicated differences in the global conformation of the two sRNAs (Figure 4A). The 3D structures showed that aCBF5 interacts non-specifically with helix P1 with positively charged residues of aCBF5 establishing electrostatic contacts and hydrogen bonds with the negatively charged ribose-phosphate backbone [37]–[39]. To deepen this analysis, we tested the importance of helix P1 for aCBF5 binding by creating two chimeric RNAs. In the first (Pab91P_1_21), helix P1 of Pab91 was replaced by that of Pab21, and in the second (Pab21P_1_91), the original P1 was replaced by that of Pab91 (Figure 5A). We observed that the presence of the P1 helix of Pab21 lowered significantly the K_D_ of aCBF5 for Pab91 into the 200 nM range (Figure 5B), and thus improved the binding of the protein to this initially poor aCBF5 binder. Interestingly, swapping of the Pab21 helix with that of Pab91 increased the K_D_ from ∼125 nM to ∼200 nM.

We next compared the CD spectra obtained with the two chimeric RNAs with those of the parental RNAs (Figure 5C). We measured λ_max_ values very close or identical for sRNAs sharing the same helix P1: sRNA Pab91 (λ_max_ = 265.6 nm) and Pab21P_1_91 (λ_max_ = 265.6 nm), and sRNA Pab21 (λ_max_ = 263.6) and Pab91P_1_21 (λ_max_ = 263.8 nm). These results confirmed that the wild-type sRNA Pab91 and Pab21 do not have the same curvature, and that helix P1 has a strong impact on the global curvature of the sRNAs. Concerning the Δε values, both are modified in the chimeric RNAs and are intermediate to the values of Pab21 and Pab91 (Δε = 8.19 M^−1^.cm^−1^ per nt for Pab21P_1_91, and 7.51 M^−1^.cm^−1^ per nt for Pab91P_1_21). Hence, the helix P1 alone cannot restore the structure of the parental sRNAs, but contributes significantly to the global nucleotide stacking.

**Figure 6:**
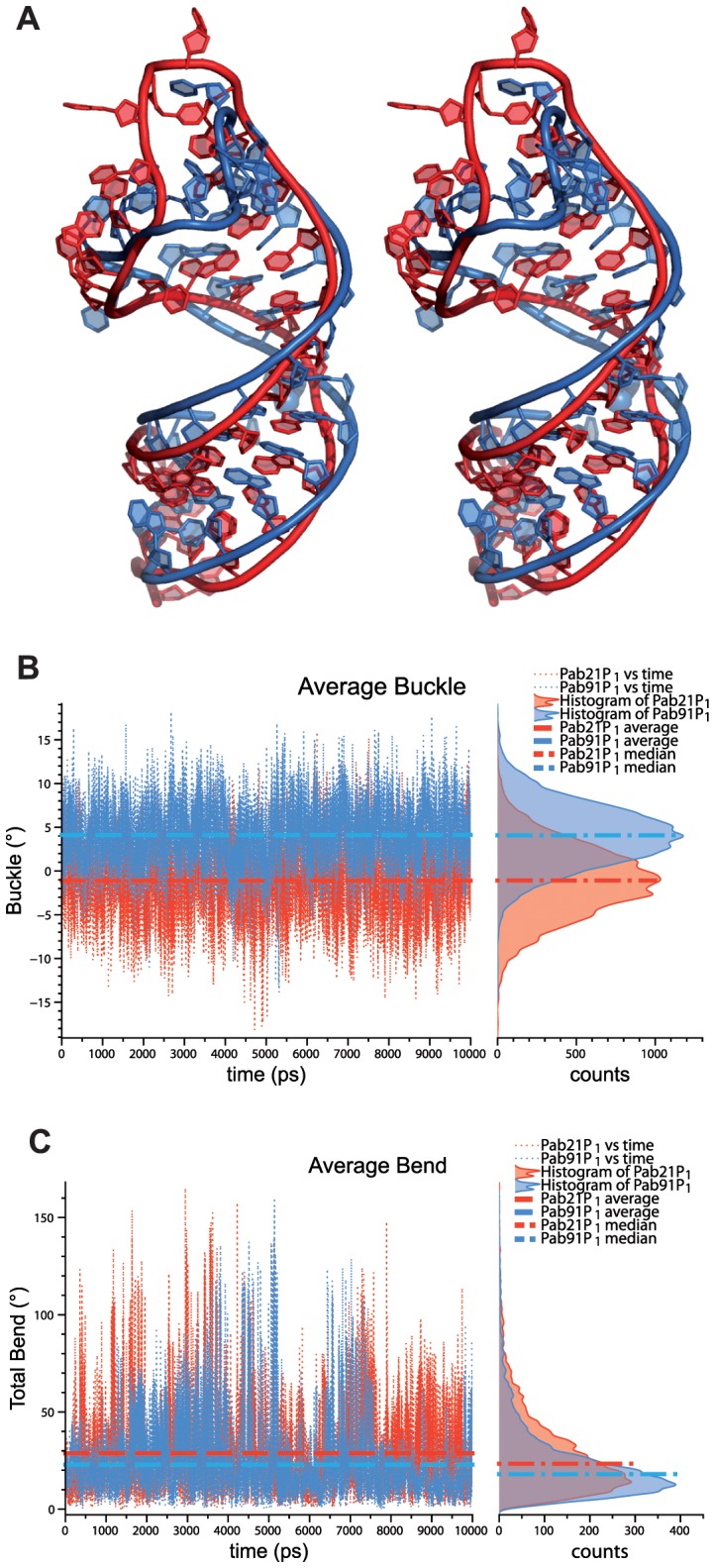
Model of the truncated forms of sRNAs Pab21 and Pab91 obtained by molecular modeling. (**A**) Stereoview of the two RNA hairpin models Pab21P_1_ and Pab91P_1_. The two RNA models are displayed using the more representative conformation (corresponding to the frame extracted from the MD trajectory which best represents the average conformation during the simulation). Pab21P_1_ (red) and Pab91P_1_ (blue) are superimposed based on the coordinates of the atoms from the phosphodiester backbone. (**B**) Average buckle versus time of the two RNA hairpin models. The plotted values are calculated by averaging the buckle on the base-pairs of helix P1 (the first base-pair of P1 is not considered in the calculations). The plots are annotated by indication of the average values of the buckle during the simulation between Pab21P_1_ (red) and Pab91P_1_ (blue). The histograms are also annotated by indication of the median values. (**C**) Average bending versus time of the two RNA hairpin models. The plotted values are calculated by averaging the bending on the nine base-pairs of P1. The plots are annotated by indication of the average values of the bent during the simulation between Pab21P_1_ (red) and Pab91P_1_ (blue). The histograms are also annotated by indication of the median values.

To improve our understanding of the relationship between the parameters of CD spectra and RNA conformation, two RNA hairpin models Pab21P_1_ and Pab91P_1_ were built (Figure 6A, respectively red and blue), corresponding to the Pab21 and Pab91 sRNAs where the terminal loop, K-loop, and upper stem were deleted and replaced by a long terminal loop (12-mer). Such truncated H/ACA sRNA were used previously to identify the structural elements which are important for protein binding; the presence of both the lower stem (including the 3′-ACA box) and a terminal loop (a dodecaloop mimicking the pseudouridylation pocket) are sufficient for binding of aCBF5 [17].

The molecular dynamics (MD) simulations performed on these two models show some subtle differences in the conformations of helix P1 associated with sequence-specific features (Figure 6A). The presence of a continuous pyrimidine tract in Pab91P_1_ (5′-CCCCUCCCC-3′) distorts the base stacking with a positive buckle at all base-pairs, while the alternation of purines and pyrimidines in Pab21P_1_ (5′-GGGCCCGGC-3′) provokes a smaller incidence of buckling, with a slight negative buckling on average, deriving essentially from the large deformation of the first base-pair of the stem, a wobble pair. We hypothesize that the more pronounced buckle (Figure 6B) would weaken the base-pair stacking in Pab91P_1_ and explain the difference in Δε_max_ measured by CD for Pab21 and Pab91. As a consequence of the buckle, the helical rise and twist slightly differ between Pab21P_1_ and Pab91P_1_ and contribute to bending more significantly Pab21P_1_ (28.7°) relative to Pab91P_1_ (22.9°) (Figures 6C and S4). These results are in agreement with the differences in λ_max_ measured between Pab21 and Pab91. Although Pab21P_1_ has a more pronounced bend, it still corresponds to a smooth circular curvature as typically found in RNA duplexes. Overall, Pab91P_1_ is more “A-like” and rigid, with a more long-lived, canonical A-form (83%) with respect to Pab21P_1_ (72%) during the time of simulation (10 ns) (Figure 6A).

### Substitution of helix P1 does not Influence RNP Activity

We next tested whether replacement of helix P1 modified the efficiency for guiding pseudouridylation (Figure 5D). Time course measurements were performed as in Figure 1D in presence of proteins L7Ae, aCBF5 and aNOP10. The chimeric RNAs Pab91P_1_21 and Pab21P_1_91 retain the ability to guide Ψ formation into their RNA substrates with an efficiency equivalent to their respective parental Pab91 and Pab21. Hence, activity of the RNP relies on a determinant distinct from helix P1, and is not always related to the efficiency of recruitment of aCBF5 on the guide sRNA.

## Discussion

### Changes in CD Spectra Associated with Changes in RNA Conformation during RNP Assembly

The data presented here show the potential of using CD in the 240−320 nm range to compare the conformation of RNAs, such as the two sRNAs Pab21 and Pab91. Importantly, this approach appears well-adapted to monitor the conformational changes occurring in RNAs upon protein binding (Figure 4). This approach requires the use of a two-chambered cell for the recording of spectra before and after mixing the contents of the two compartments. This procedure allowed us to follow the dynamics of RNA conformation during a step-by-step addition of proteins to assemble an RNA–multiprotein complex.

Our data show that the core proteins have different impacts on the conformation of the guide RNAs during the stepwise assembly of the box H/ACA sRNP LCN. Binding of L7Ae to the Pab21 sRNA has a low impact on the global conformation of the guide sRNA. The slight effect on RNA base stacking (reflected by the CD signal Δε) likely occurs in the K-turn motif, which is folded upon L7Ae binding [53]. The higher values of the CD signal λ_max_ observed upon aCBF5 binding indicate a modification in the global conformation of Pab21 − most likely a change in its curvature. This effect was not a general feature for all sRNAs, however, as we did not detect modification in the λ_max_ value upon binding of aCBF5 to Pab91 (data not shown). In the archaeal sRNP H/ACA, the PUA domain of aCBF5 is anchored to the lower part of the sRNA; the stability of the aCBF5–sRNA complex also arises from contacts between the catalytic domain and helix P2, whose basal region is wrapped around the catalytic domain in the structure of the H/ACA sRNP [37], [40]. The contacts established between aCBF5 and the sRNA within the aCBF5–sRNA complex persist during RNP assembly, as most of them are conserved within the H/ACA sRNP [50]. The major conformational effects linked to aCBF5 sRNA binding could arise from a modification of the tertiary structure of helix P2 and/or helix P1 (see last section).

Addition of aNOP10 to the aCBF5–sRNA complex substantially influences the sRNA conformation, as evidenced by both an increase in λ_max_ and a reduction of Δε for both Pab21 and Pab91 (Figure 3). Nevertheless, such a conformational change is not sufficient for optimal activity, as the aCBF5–aNOP10–sRNA sub-sRNP achieves only a low rate of modification (Figure 1C and [18]). A crystal structure of a sRNP containing aNOP10 but lacking both a K-turn motif and the protein L7Ae, showed that helix P2 adopts a different orientation to that in the fully-assembled sRNP [40]. This sub-RNP can bind substrate RNA, but in an inactive conformation [40]. Improper orientation of helix P2 and misplacement of the substrate RNA were proposed to result from the absence of protein L7Ae [40]. In agreement with this hypothesis, we show that addition of L7Ae to the sub-RNP aNOP10–aCBF5–Pab21 lead to an additional sRNA conformational change, with modification in the CD signals (Figures 3 and 4). Importantly, since this conformational change was not detected when the interaction between aNOP10 and L7Ae was impaired (Figure 4E), it is likely that in this variant sub-RNP, the sRNA is not locked in the bent conformation found in the crystal structure of box H/ACA sRNP [37].

### The Respective Contributions of L7Ae and aNOP10 to sRNP Activity

As the activity of sRNPs Pab21 and Pab91 containing L7Ae, but in which no aNOP10–L7Ae interaction is possible, is only slightly higher than those of sRNPs lacking L7Ae (LCN-Y41A/Y44A vs. CN in Figure 1D), we propose that for the majority of the sRNAs, the positive effect of L7Ae on sRNP activity depends on its interaction with aNOP10. This statement is in accord with a change in the substrate conformation (Figure 2), and the low amounts of stable substrate RNA–sRNP complexes (designated CII′) observed in the absence of the aNOP10–L7Ae interaction for the two sRNAs (Figure 1F and G).

Binding of L7Ae to the apical K-turn motif *per se* has been proposed to indirectly influence substrate RNA binding by remodeling the sRNA pseudouridylation pocket [26]. This hypothesis was based on data obtained by probing binding of L7Ae on the *P. furiosus* sRNA Pf9. We observed a stronger accessibility to lead-in strand s1 of the Pab21 pseudouridylation pocket upon L7Ae binding (Figure 3A, lane 4). However, we did not observe any changes in the pattern of lead cleavages occurring in strands s1 and s2 of the L7Ae-Pab91 sRNA sub-complex (Figure 3B, lane 4), suggesting that binding of L7Ae to sRNA *per se* had no detectable effect on the folding of the pseudouridylation pocket. This observation reinforces the proposal that the major effect seen on RNA base stacking by CD measurement is confined to the K-turn motif. We investigated whether folding of the pocket was also unaffected within sub-complexes formed between L7Ae and Pab160, the functional homolog of Pf9 in *P. abyssi,* as well as with another *P. abyssi* sRNA Pab19 (Figure S3). Interestingly, only binding of L7Ae to Pab160 lead to more extensive cleavage in strand s2 (Figure S2, lane 15). Hence, remodeling of the pseudouridylation pocket is not a general feature of the binding of L7Ae to a guide sRNA.

It remains to be determined whether the interaction of aNOP10 with L7Ae is a conserved feature of the box H/ACA RNA guided system. Unlike L7Ae, human NHP2 is found associated with the dimer Dyskerin(CBF5)-NOP10 in absence of the guide RNA [54], but the precise mode of interaction is not known. Recent 3D structures obtained for yeast protein components suggest a similar positioning of Nhp2p within the snoRNPs as in sRNPs [55], [56], despite the absence of K-turn or K-loop motifs in the H/ACA snoRNAs. The three residues in helix α3 are not strictly conserved in L7Ae homologs in yeast (H89, L93, D96 in Nhp2) and human (H93, M97, D100 in NHP2) but might account for a similar interaction with Nop10p/NOP10. During H/ACA snoRNP assembly, the Dyskerin(CBF5)–NOP10–NHP2 complex formed in the cytoplasm is then loaded by NAF1 on the nascent snoRNAs in the nucleus [57]. This current model raises the question of whether the interactions between NOP10 and NHP2 found in the archaeal particle are formed, and if so, whether they occur upon eukaryotic snoRNP assembly or preexist in the RNA unbound state of the Dyskerin(CBF5)–NOP10–NHP2 protein complex.

### The Strength of aCBF5 Binding to P1 cannot Predict Catalytic Activity

The starting point of this study was the distinct affinities of the core protein aCBF5 and the heterodimer aCBF5–aNOP10 for the two box H/ACA sRNAs Pab21 and Pab91. In the conditions used for EMSA and despite the very low affinity of aCBF5 for Pab91, a stable complex CII is obtained with this sRNA in the presence of its substrate RNA. The presence of the substrate RNA likely displaces the equilibrium towards complex formation by enhancing the thermodynamic stability of the LCN RNP. The CII complex shows the pattern of lead cleavages, which is obtained readily upon proteins binding on Pab21 to form complex LCN (Figure 3). Characterization of the chimera resulting from a P1 swap between Pab21 and Pab91 showed that the basal helix P1 is a key determinant explaining the differences in the affinity of aCBF5 for the sRNA. The slight differences in the conformations of the two RNA hairpin models during the simulations suggest that Pab21P_1_ is more prone to be bent upon aCBF5 binding and would facilitate the RNP assembly. However, the conformation of P1 in the fully assembled RNP complex (including the RNA guide and RNA substrate) is very close to a canonical A-form, where the substrate should be in some optimal binding mode for catalysis (PDB ID: 3LWR) [58]. On the other hand, once the RNP particle is formed, the more rigid and “A-like” Pab91 would facilitate the positioning of the substrate in the catalytic pocket, and explain the higher activity of Pab91 sRNP. Although the assembly of the RNP particle on Pab91P_1_21 is less efficient than Pab21, the more rigid P1 helix can thus contribute to increase the catalytic efficiency (Figure 5D). Nevertheless, the presence of helix P1 of Pab91 in the chimera Pab21P_1_91 does not enhance activity of the RNP toward an efficient modification of the Pab21 substrate RNA. These data strongly suggest that other regions of Pab21, and likely the pseudouridylation pocket, are not optimized for substrate modification once the RNP is assembled. One possibility might be that the substrate of Pab21 could associate with the RNP through two modes of base-pairing [19], one being active and one non-active, which would titrate the Pab21 sRNP activity. This would explain that the activity plateaus at ∼50% modification of the substrate (Figure 1D).

## Supporting Information

Figure S1 **Effect of mutations in protein aNOP10 on the rate of Ψ55 modification by aCBF5 in tRNA.** (**A**) Secondary structure models of *P. abyssi* tRNA^Asp^. Residue U55 is circled. The CCA sequence at the 3′ end is boxed. The CCA deletion in variants tRNA^Asp^-ΔCCA is shown. (**B**) Time course analysis of Ψ55 formation in tRNA. The tRNA^Asp^−ΔCCA substrate was radiolabeled during *in vitro* transcription by incorporation of [α−^32^P]CTP. It was incubated at 65°C with the protein set aCBF5–aNOP10 (CN). A mutant of protein aNOP10 Y41A/Y44A (Y) was used for the reaction. After T2 RNase digestion, the amount of Ψ formation was estimated by 2D-TLC analysis.
(TIF)

Figure S2 **Effect of mutations in proteins L7Ae and aNOP10 on substrate RNA positioning within the sRNP.** The fluorescence intensity at 366 nm was monitored while the substrate RNA labeled with both 5-FU and 2-AP bound with the Pab21 guide RNA was first titrated with saturating amounts of the sRNP proteins. All proteins were present at 5× molar excess relative to the RNA to ensure full binding. Comparison of the fluorescence intensity profiles of titration by the wild type proteins (dark blue trace) to the profiles recorded with mutant proteins aNOP10 Y41A/Y44A (green trace) (**A**), and with L7Ae H70A/L74A/E77A (pink trace) (**B**). The arrows indicate the time points at which each specific protein was added, where CN denotes the aCBF5–aNOP10 complex, CY denotes the aCBF5–aNOP10 Y41A/Y44A mutant complex, L denotes L7Ae and HLE denotes the L7Ae mutant H70A/L74A/E77A.
(TIF)

Figure S3 **Chemical probing of sub-complexes formed by the association of L7Ae with various sRNA.** (**A** and **C**) Secondary structure models of *P. abyssi* Pab19 and Pab160 sRNAs. (**B** and **D**) Footprinting of protein L7Ae (L) on the various sRNAs. Reactions with lead were carried out on 5′-^32^P end-labeled sRNA as in Figure 3. Samples were fractionated on 10% polyacrylamide denaturing gels containing 8 M urea. Lane OH^−^ and T1 correspond, respectively, to an alkaline hydrolysis ladder, and RNase T1 digestion ladder obtained under denaturing conditions.
(TIF)

Figure S4 **Average h-Twist versus time of the two RNA hairpin models Pab21P_1_ and Pab91P_1_.** The plotted values are calculated by averaging the helical twist on the nine base-pairs of P1. The plots are annotated by indication of the average values of the buckle during the simulation between Pab21P_1_ (red) and Pab91P_1_ (blue). The histograms are also annotated by indication of the median values.
(TIF)
