# Supplementary figures and images for: Comparative Study of Two Box H/ACA Ribonucleoprotein Pseudouridine-Synthases: Relation between Conformational Dynamics of the Guide RNA, Enzyme Assembly and Activity
Source: PLoS One. 2013 Jul 29;8(7):e70313. doi: 10.1371/journal.pone.0070313 (PMC3726423; doi:10.1371/journal.pone.0070313)

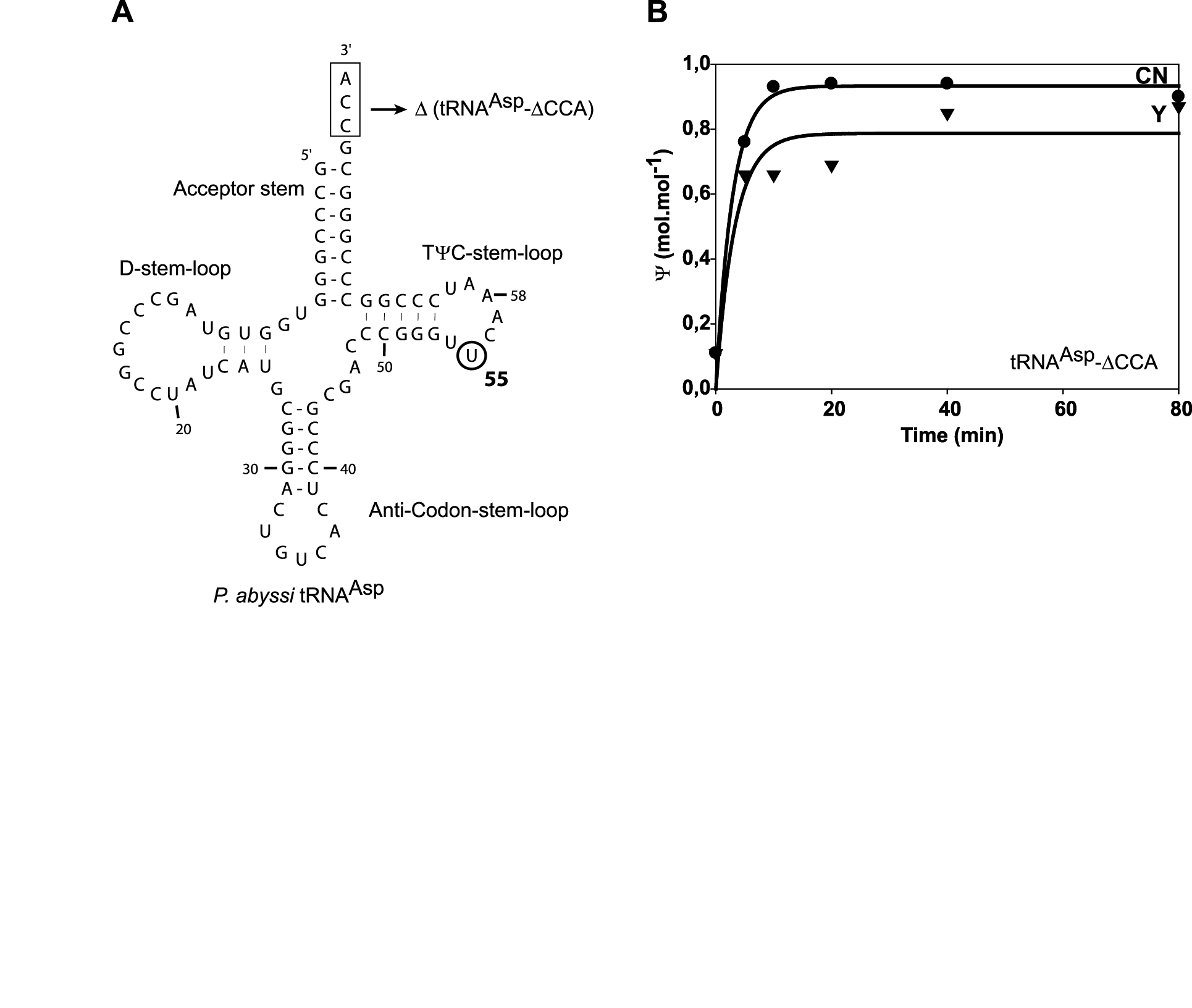

Supplement: Figure S1 — Effect of mutations in protein aNOP10 on the rate of Ψ55 modification by aCBF5 in tRNA. (A) Secondary structure models of P. abyssi tRNAAsp. Residue U55 is circled. The CCA sequence at the 3′ end is boxed. The CCA deletion in variants tRNAAsp-ΔCCA is shown. (B) Time course analysis of Ψ55 formation in tRNA. The tRNAAsp−ΔCCA substrate was radiolabeled during in vitro transcription by incorporation of [α−32P]CTP. It was incubated at 65°C with the protein set aCBF5–aNOP10 (CN). A mutant of protein aNOP10 Y41A/Y44A (Y) was used for the reaction. After T2 RNase digestion, the amount of Ψ formation was estimated by 2D-TLC analysis. (TIF) [file pone.0070313.s001.tif]

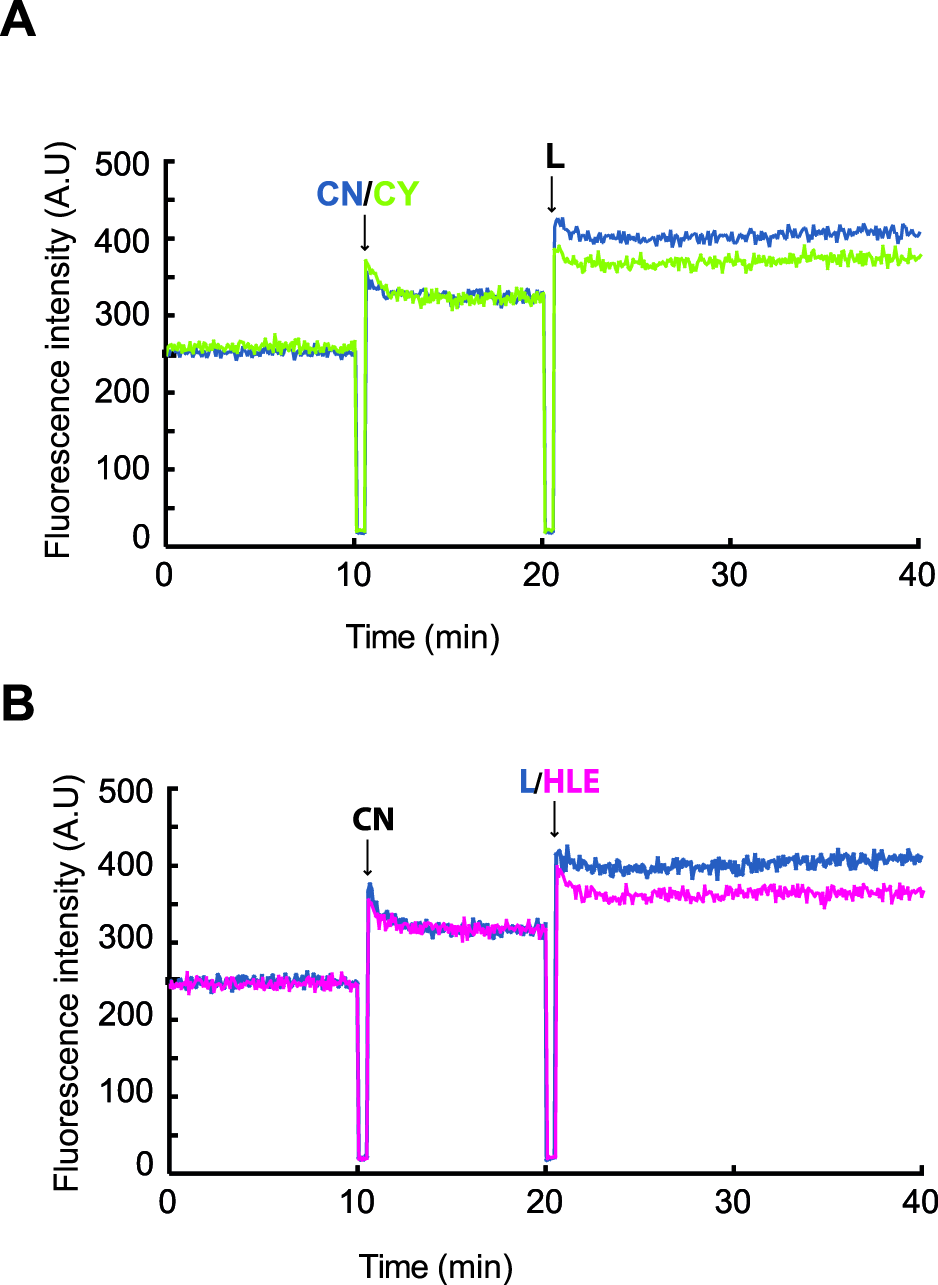

Supplement: Figure S2 — Effect of mutations in proteins L7Ae and aNOP10 on substrate RNA positioning within the sRNP. The fluorescence intensity at 366 nm was monitored while the substrate RNA labeled with both 5-FU and 2-AP bound with the Pab21 guide RNA was first titrated with saturating amounts of the sRNP proteins. All proteins were present at 5× molar excess relative to the RNA to ensure full binding. Comparison of the fluorescence intensity profiles of titration by the wild type proteins (dark blue trace) to the profiles recorded with mutant proteins aNOP10 Y41A/Y44A (green trace) (A), and with L7Ae H70A/L74A/E77A (pink trace) (B). The arrows indicate the time points at which each specific protein was added, where CN denotes the aCBF5–aNOP10 complex, CY denotes the aCBF5–aNOP10 Y41A/Y44A mutant complex, L denotes L7Ae and HLE denotes the L7Ae mutant H70A/L74A/E77A. (TIF) [file pone.0070313.s002.tif]

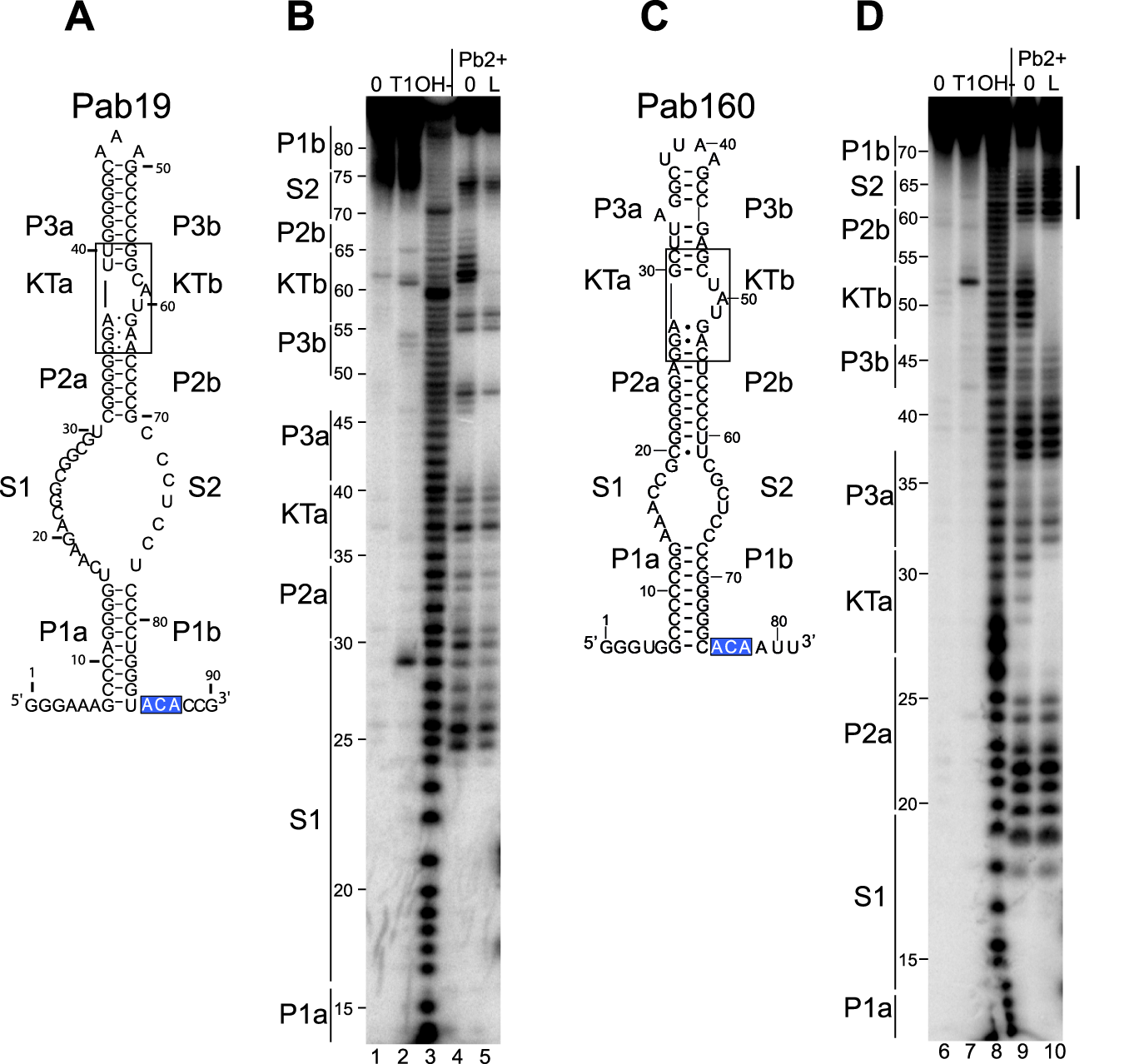

Supplement: Figure S3 — Chemical probing of sub-complexes formed by the association of L7Ae with various sRNA. (A and C) Secondary structure models of P. abyssi Pab19 and Pab160 sRNAs. (B and D) Footprinting of protein L7Ae (L) on the various sRNAs. Reactions with lead were carried out on 5′-32P end-labeled sRNA as in Figure 3. Samples were fractionated on 10% polyacrylamide denaturing gels containing 8 M urea. Lane OH− and T1 correspond, respectively, to an alkaline hydrolysis ladder, and RNase T1 digestion ladder obtained under denaturing conditions. (TIF) [file pone.0070313.s003.tif]

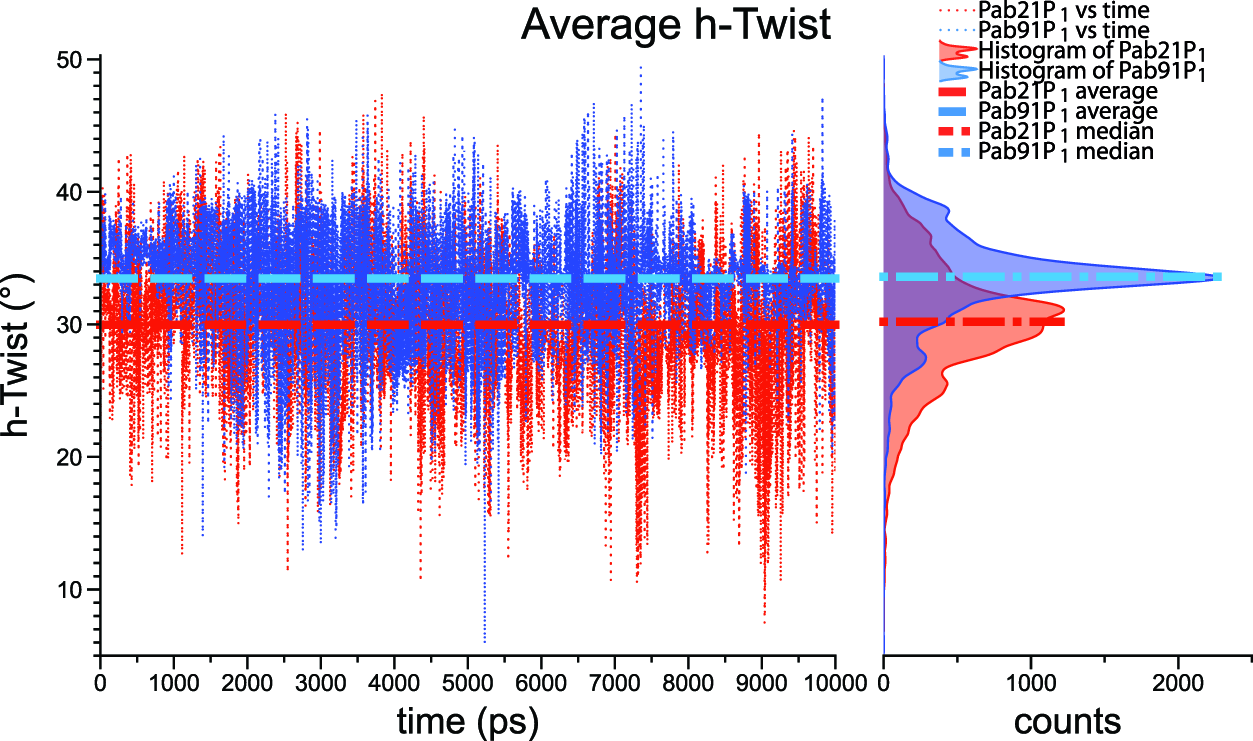

Supplement: Figure S4 — Average h-Twist versus time of the two RNA hairpin models Pab21P1 and Pab91P1. The plotted values are calculated by averaging the helical twist on the nine base-pairs of P1. The plots are annotated by indication of the average values of the buckle during the simulation between Pab21P1 (red) and Pab91P1 (blue). The histograms are also annotated by indication of the median values. (TIF) [file pone.0070313.s004.tif]
